# Model sensitivity limits attribution of greenhouse gas emissions to polar bear demographic rates

**DOI:** 10.1038/s41598-025-89218-3

**Published:** 2025-02-10

**Authors:** Ryan R. Wilson, Erik M. Andersen

**Affiliations:** U.S. Fish and Wildlife Service, Marine Mammals Management, Anchorage, AK USA

**Keywords:** Climate attribution, Climate change, Greenhouse gas emissions, Endangered species act, Polar bears, *Ursus maritimus*, Climate-change ecology, Conservation biology, Ecological modelling, Population dynamics, Marine biology

## Abstract

Greenhouse gas emissions continue to increase and negatively affect sea ice conditions that polar bears rely on. It is therefore important to better understand how specific emissions levels affect polar bear demography. A recent study proposed a framework to address this issue, but sensitivity to decisions rules of the approach may limit its utility. We tested how sensitive the approach is to decisions rules related to sea ice concentration, choice of subpopulation boundaries, and modeling choices for bears in the Chukchi Sea and Southern Beaufort Sea subpopulations. We found that the number of ice-free days, number of fasting days, and when 10% of reproductive females exhibited recruitment failure varied considerably depending on equally-valid decisions rules versus those used in the existing study. Whereas the previous study suggested that both subpopulations surpassed the critical number of ice-free days that negatively affect recruitment, we found this threshold was never reached by the Southern Beaufort Sea subpopulation and only once for the Chukchi Sea subpopulation for the decision rules we considered. Our results suggest that the previously published approach is too sensitive to modeling assumptions and choice of decision rules to accurately evaluate the impacts of GHG emissions on polar bear demographic rates.

**Supplementary Information:**

The online version contains supplementary material available at 10.1038/s41598-025-89218-3.

## Introduction

Climate change is a significant force reshaping ecosystems^1^, communities^2^, and populations^3^ globally. No species has received as much attention about the impacts of climate change as polar bears^4^ (*Ursus maritimus*), which were the first species specifically listed under the Endangered Species Act due to concerns about declines in their sea ice habitat directly resulting from climate change^5^. Research has confirmed on multiple occasions that sea ice loss resulting from climate change is the primary threat to the long-term persistence of the species^6–8^.

Reductions to their sea ice habitat has led to more polar bears coming on shore in summer and autumn^9,10^ and staying there for longer^11^. With few exceptions (e.g., whale remains^12^), terrestrial food resources are not thought to provide sufficient energy to polar bears to compensate for increased periods of fasting while on shore^13^. Other studies have shown that polar bears in some subpopulations are also showing increased fasting during spring^14^ when bears should be maximizing energetic gains prior to summer and autumn when hunting becomes less successful due to sea ice melting or retreating over the less productive waters of the Arctic Basin^15^. Thus, there is concern that decreased energetic gains, and increased periods of fasting in summer and autumn may lead to negative demographic impacts for polar bears^16^, ultimately leading to population declines.

Current levels of sea ice loss have led to declines in some polar bear subpopulations^17,18^ and numerous studies have established likely linkages between sea ice loss and polar bear demographics^18–20^. It has become clear, however, that not all polar bear subpopulations are responding to sea ice loss similarly. This was highlighted by a comparative study of how recruitment and body conditions of bears have changed through time in the adjacent Chukchi Sea (CS) and Southern Beaufort Sea (SBS) subpopulations^20^. Both have experienced significant declines in summer sea ice, yet the CS subpopulation has not exhibited the declines in recruitment or body condition that have been observed in the adjacent SBS subpopulation^19,20^. These results imply that the response of a polar bear subpopulation to climate change is not simply related to the level of sea ice loss.

The ability to determine the level of cumulative greenhouse gas (GHG) emissions that could lead to negative demographic impacts to polar bear subpopulations has, to date, eluded researchers. A recent study by Amstrup and Bitz^21^, however, developed an approach to link the volume of cumulative GHG emissions to reductions in polar bear recruitment. Because the relationship between polar bear energetics and GHG emissions has not yet been established, Amstrup and Bitz^21^ relied on metrics (e.g., number of fasting days) derived from sea ice concentration data^22^ to serve as indices of this relationship. They then used their derived fasting days to link polar bear demographic data^16^ to cumulative GHG emissions (i.e., CO_2_-equivalent^21^; see Supplemental Methods S1).

While Amstrup and Bitz^21^ suggested numerous factors that likely need to be addressed before their approach can be effectively applied (e.g., body mass of bears when they first arrive on shore), several critical assumptions were not acknowledged that could significantly affect the utility of their approach. Specifically, the decision rules related to sea ice concentration that serve as the foundation of the Amstrup and Bitz^21^ approach might not be appropriate across all polar bear subpopulations as is implicitly assumed within the analysis. Further, Amstrup and Bitz^21^ analyzed data according to subpopulation boundaries identified in Amstrup et al.^6^. These boundaries, however, are not universally applied and other studies have relied on different delineations that more accurately reflect the biological conditions encountered by subpopulations (e.g., Rode et al.^23^). Finally, when attempting to relate the number of fasting days with recruitment failure, Amstrup and Bitz^21^ assume a linear relationship when a logistic-type curve may be a better fit (Figure S2a in Amstrup and Bitz^21^). Each of these factors has the potential to significantly alter the results of Amstrup and Bitz^21^. There is therefore a need to better understand how sensitive their approach is to reasonable alternatives to these three factors.

In this study, we sought to better understand sources of uncertainty in the Amstrup and Bitz^21^ framework that may make it difficult to attribute levels of cumulative GHG emissions to polar bear demographic impacts. The focus of our analysis is on parameters and decision rules that were not discussed by Amstrup and Bitz^21^ that need further research before their approach can be viably applied. Specifically, we consider how sensitive inferences from their approach are to different definitions of a subpopulation’s boundary, decision rules on what constitutes an ice-free day and fasting days, and finally modeling the relationship between fasting days and recruitment failure as a non-linear vs. linear relationship. Because Amstrup and Bitz^21^ recommended that their analysis be applied to Endangered Species Act evaluations, we restrict our analysis to the SBS and CS subpopulations, which are the only subpopulations within the jurisdiction of the United States.

## Methods

We used the same sea ice concentration dataset^22^ and methods detailed in Amstrup and Bitz^21^ for calculating sea ice extent and ice-free days (see Supplemental Methods S1). Because we were interested in identifying how sensitive the framework developed by Amstrup and Bitz^21^ is to key decision rules, we considered deviations to their approach for four factors: definition of sea ice extent, reference threshold for calculating ice free days (IFD), definition of subpopulation boundary, and modeling the relationship between fasting duration (FD) and recruitment failure (RF) based on data obtained from Molnár et al.^16^. We detail these deviations below.

### Sea ice extent

Although sea ice is the basis of polar bear habitat and metrics related to sea ice are regularly included in ecological models for polar bears, there is no metric that has been applied universally. This is likely due to variation in sea ice dynamics across subpopulations which ultimately led Amstrup et al.^6^ to classify polar bear subpopulations into four different ecoregions based on how they interact with sea ice in their ranges.

Sea ice extent, has been linked to multiple aspects of polar bear demography, but there is no consensus in how it is defined across subpopulations or studies. Amstrup and Bitz^21^ defined sea ice extent as the total area within a subpopulation boundary where ice concentration was > 30%. While previous studies have used 30% sea ice concentration (SIC) to define productive ice for polar bears (e.g., Cherry et al.^24^), others have used lower SIC thresholds related to timing of arrival onshore^9,10^. For example, a study of polar bears in the SBS subpopulation^9^ found that 15% SIC was predictive of when polar bears come to shore at the beginning of autumn (although they only considered 15% and 50% SIC). Another study on the CS subpopulation also found a relationship between how long polar bears spend on land in autumn and sea ice extent defined by SIC > 15%^10^. Thus, there is no well-established value of SIC that defines sea ice extent in a manner biologically relevant to polar bears. We note, however, that these studies^9,10^ did not consider if 30% SIC was more predictive than 15%, but that researchers for these study systems believed 15% SIC was an appropriate choice and found support for it with their data.

The differences in how bears interact with sea ice across subpopulations^6^ make it unlikely that a single SIC value is suitable for all subpopulations. This point is further highlighted by a recent study that demonstrated that bears in the SBS subpopulation did not exhibit a large increase in departure from the ice towards shore in summer until SIC was < 15%^25^. Interestingly, when Kellner et al.^25^ compared the results from the SBS subpopulation with the Western Hudson Bay (WHB) subpopulation, they found that bears from the WHB began moving to shore in higher numbers when SIC was < 30%, consistent with the study by Cherry et al.^24^ which Amstrup and Bitz^21^ used to justify their approach. Kellner et al.^25^ hypothesized that because bears in the WHB subpopulation live in the seasonal ice ecoregion, they are accustomed to coming to shore and thus begin their shoreward migration at higher ice concentrations. Conversely, until recently, polar bears in the SBS subpopulation typically spent the entire year on ice, so might be more likely to stay on sea ice as long as possible before moving to shore.

Given variation in the concentration of sea ice used by polar bears in different parts of their range and that the SBS and CS subpopulations occur in a different ecoregion than the WHB subpopulation, using a single value to represent sea ice extent is unlikely to be most appropriate for all polar bear subpopulations. Because multiple studies identified polar bear use of sea ice concentrations as low as 15%, we considered how results of the Amstrup and Bitz^21^ model would change if sea ice extent was defined by a 15% SIC threshold versus the 30% SIC threshold used in their study.

### Ice-free days

To link changes in sea ice to polar bear energetics, and ultimately recruitment, Amstrup and Bitz^21^ assumed that an IFD is equivalent to a FD (i.e., a day when ice conditions are not suitable for bears to capture prey). They defined an IFD as a day when sea ice extent (as defined above, often referred to as sea ice area by others, e.g., Rode et al.^23^) was < 30% of the mean sea ice extent in March between 1979 and 1988^21^. Similar to the 30% threshold of SIC used to define ice extent, the choice of a 30% area threshold to identify IFD is not well-established in the literature, nor is the method for establishing the critical threshold (i.e., using the 10-year March average between 1979 and 1988). For example, Hamilton et al.^26^ defined the “ice-free” period for polar bears as the annual duration of time between when SIC first drops below 50% until freeze-up begins (i.e., SIC rises above 10%). Another study attempting to standardize sea ice metrics for polar bears globally defined an IFD as any day where sea ice extent dropped below the midpoint between the mean sea ice extents in March and September each year^27^.

Because an energetics model is not used by Amstrup and Bitz^21^ to define a FD but instead is based on an index derived from sea ice extent, the choice of which threshold is used to define a FD could lead to significant changes in results. To explore this sensitivity, we considered how the results of Amstrup and Bitz^21^ would change if we defined FD as any day where sea ice extent dropped below 15% of the average March extent between 1979 and 1988. Similar to the 30% threshold used by Amstrup and Bitz^21^, we recognize that our choice of a 15% threshold to define FDs is also not well-established in the literature. We wanted to determine, however, how the Amstrup and Bitz^21^ approach would differ if the critical IFD was significantly less than they used given observations of significant sea ice loss in the Chukchi Sea and the long-term stability of the CS subpopulation^20^.

### Subpopulation boundaries

**Fig. 1:**
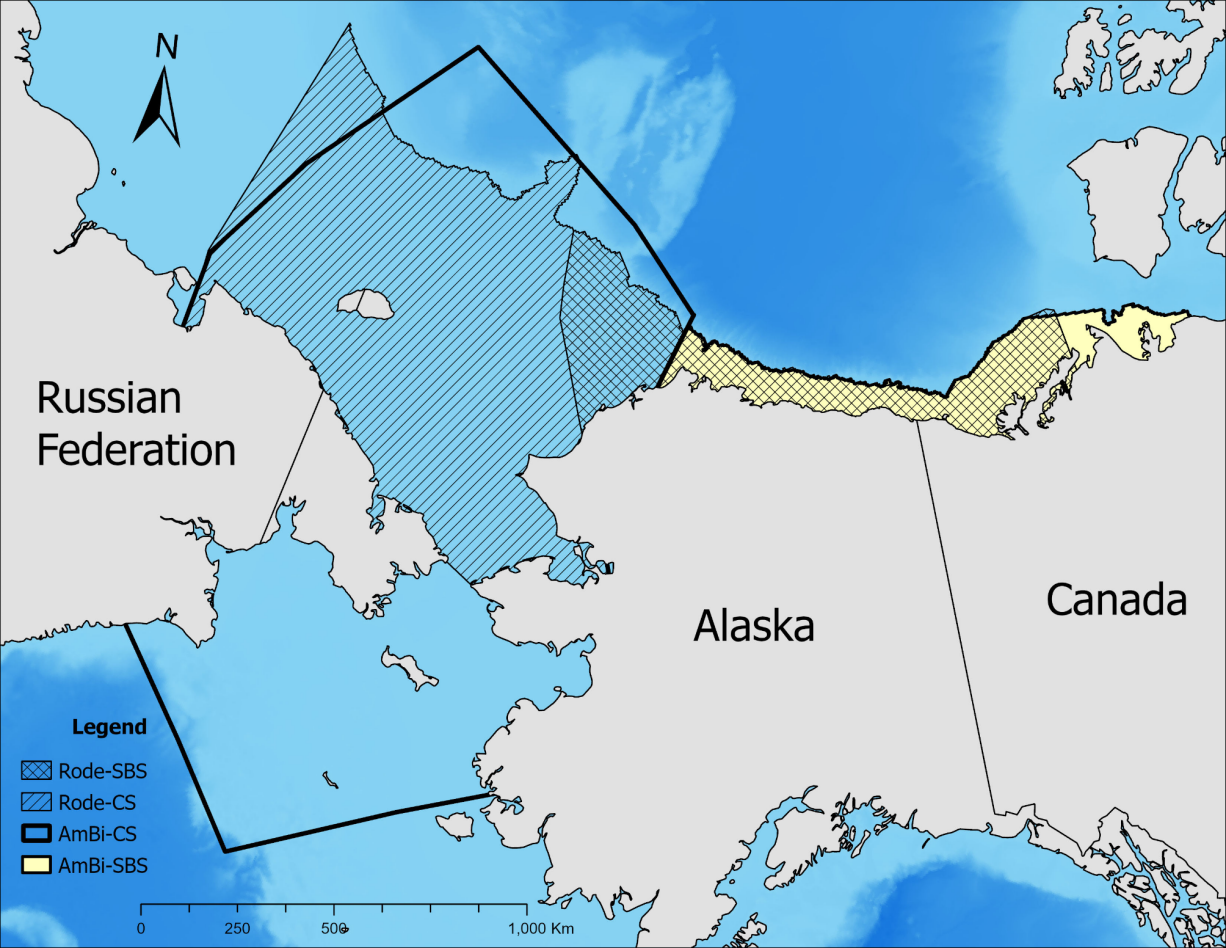
Subpopulation boundaries for the Southern Beaufort Sea (SBS) and Chukchi Sea (CS) as defined in Amstrup and Bitz^21^ (AmBi) and Rode et al.^23^ (Rode). This map was created with ArcGIS Pro version 3.3 (https://www.esri.com/en-us/arcgis/products/arcgis-pro/overview).

Objectively delineating polar bear subpopulation boundaries is an inherently difficult task given the lack of clear barriers to movements between subpopulations (e.g., Scharff et al.^28^). This is especially true for the SBS and CS subpopulations. The Polar Bear Specialist Group (PBSG) is typically recognized as the entity that formally designates these boundaries. Rather than use the formal PBSG boundaries, however, numerous researchers have restricted their delineations to the regions receiving regular use by polar bears based on an extensive collection of collar locations^23,29^ (Fig. 1). The boundaries used for the CS and SBS subpopulations by Amstrup and Bitz^21^ do not represent the formal PBSG boundaries but instead rely on modifications as presented in Amstrup et al.^6^. Additionally, for the SBS subpopulation specifically, Amstrup and Bitz^21^ restricted their analysis to the portion of the SBS range that occurs over shallow continental shelf water (i.e., < 300 m depth). A similar restriction for the CS has been used by others^23,29^ given the low use of the area south of the Bering Strait, and Rode et al.^20^ found that the period of ice cover over the continental shelf was a key factor leading to observed differences in how the subpopulations have responded to sea ice loss.

Because the approach presented by Amstrup and Bitz^21^relies on sea ice conditions to define FD, it could be sensitive to the choice of subpopulation boundary used, especially given that the distribution of shallow, productive waters can vary across a subpopulation’s range (e.g., SBS^20^). We therefore considered how the results of Amstrup and Bitz^21^ would differ if different subpopulation boundaries were used for the CS and SBS. Specifically, we used the CS-shelf and SBS-shelf boundaries as defined by Rode et al.^23^ to represent the ranges of the subpopulations for analysis and to determine how sensitive the results of Amstrup and Bitz^21^ are to boundary choice.

### Linear versus non-linear model

**Fig. 2:**
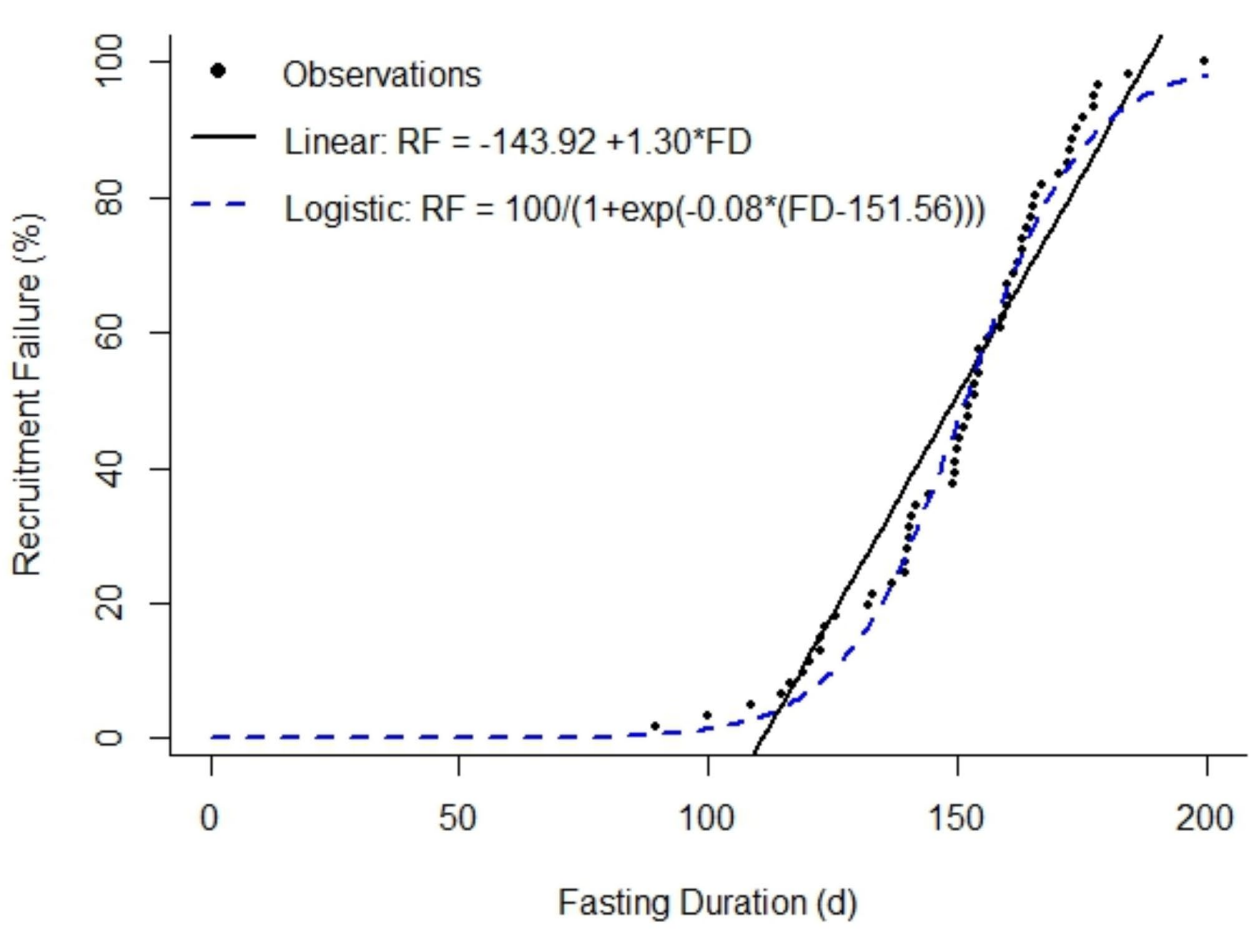
Two different model fits to relate the fasting duration of polar bears to the percent of reproductive females that exhibit reproductive failure. Observed data are from Molnár et al.^16^ and were collected from polar bears in the Western Hudson Bay subpopulation. The solid black line represents the estimated linear regression fit to the data. The hashed-blue line represents the estimated logistic regression curve fit to the data. The linear regression allows impossible values below 0 and above 100 whereas the fitted logistic curve restrains fitted values within the bounds supported by the underlying process.

The central feature of the Amstrup and Bitz^21^ approach that allows for a link between GHG emissions and polar bear recruitment failure (RF; represented as the percent of females with recruitment failure) is their integration of coefficient estimates from the relationship between GHG and FD with coefficient estimates from the relationship between FD and RF (based on data from Molnár et al.^16^). Specifically, they estimate a linear relationship between GHG and FD: $$\:FD=\:{\gamma\:}_{0}+\:{\gamma\:}_{1}GHG$$ where ***γ*** are the modeled regression coefficients. They then estimate a linear relationship between FD and RF: $$\:RF=\:{\beta\:}_{0}+\:{\beta\:}_{1}FD$$, where ***β*** are the modeled regression coefficients. They then substitute the linear regression from the first model for FD in the second regression to establish RF as a function of GHG: $$\:RF=\:$$$$\:{\beta\:}_{0}+\:{\beta\:}_{1}(\:{\gamma\:}_{0}+\:{\gamma\:}_{1}GHG)$$. The relationship between FD and RF, however, is clearly non-linear, appearing to have a sigmoidal shape (Fig. 2, and see Fig. S2 in Amstrup and Bitz^21^). Treating this relationship as linear leads to biologically nonsensical estimates of RF, with percentages < 0% for low levels of cumulative GHG and > 100% for higher levels of cumulative GHG (see Fig S2 in Amstrup and Bitz^21^).

We therefore considered how the results from Amstrup and Bitz^21^ would change if the relationship between FD and RF was modeled as a logistic curve rather than linear (Fig. 2). We used the ‘nls’ function in program R^30^ to model the relationship between FD and RF as: $\:RF=\:100/\left(1+\text{e}\text{x}\text{p}\left(-r\left(FD-{x}_{0}\right)\right)\right)$ where 100 represents the maximum value RF can take (i.e., 100%), *r* is the logistic growth rate, and $\:{x}_{0}$ is FD value at the mid-point of the logistic curve. We then integrated the results from regression between GHG and FD into the logistic equation to obtain a logistic curve that related GHG to RF: $\:RF=\:100/\left(1+\text{e}\text{x}\text{p}\left(-r\left({\gamma\:}_{0}+\:{\gamma\:}_{1}GHG\:-{x}_{0}\right)\right)\right)$.

### Analysis

Following the general methods of Amstrup and Bitz^21^, we first calculated the reference extents for the different combinations of ice-free day thresholds, sea ice extent thresholds, and boundaries (Table 1). Based on these reference extents, we then calculated the number of IFD for the SBS and CS subpopulations for each unique combination of SIC (i.e., 15 vs. 30%), IFD threshold (i.e., 15 vs. 30%), and boundary definition (i.e., Amstrup et al.^6^ vs. Rode et al.^23^) leading to 8 unique sets of IFD estimates for each subpopulation. We then compared these results to the definitions Amstrup and Bitz^21^ used to identify IFD (i.e., 30% SIC, 30% IFD threshold, Amstrup et al.^6^ boundaries). As in Amstrup and Bitz^21^, we also identified the year in which the recruitment impact threshold was crossed. We followed Amstrup and Bitz^21^ in defining the recruitment impact threshold as the first year when the number of annual IFD was above 117 for 3 out of 5 years. It is worth restating, however, that this value was derived for the WH subpopulation and may not be representative for either the CS or SBS.

**Table 1:** Reference subpopulation extents (km^2^) based on mean sea ice extent in March between 1979–1988 for two different values of sea ice concentration used to define sea ice extent (i.e., < 15% or < 30% sea ice concentration) and two different definitions of the threshold used to define an ice-free day (IFD; i.e., < 15% or < 30% of 10-year March average sea ice concentration).

Subpopulation	Sea ice concentration (%)			
	15		30	
	IFD threshold (%)		IFD threshold (%)	
	15	30	15	30
Chukchi Sea – AmBi	2140.0	4279.9	2117.1	4234.2
Chukchi Sea – Rode	1122.8	2245.6	1122.8	2245.6
Southern Beaufort Sea – AmBi	212.5	424.9	212.5	424.9
Southern Beaufort Sea – Rode	318.2	636.4	318.2	636.4

Subpopulation boundary is identified by ‘AmBi’ or ‘Rode’ to indicate boundary definitions defined by Amstrup and Bitz^21^ and Rode et al.^23^, respectively.

Based on these sets of IFD, we estimated regression coefficients for the relationship between the annual cumulative GHG emissions obtained from Amstrup and Bitz^21^ and our calculated IFD each year. For each set of definitions for each subpopulation, we then calculated the cumulative GHG emissions level required to result in a 10% increase in RF for both the linear relationship used by Amstrup and Bitz^21^ and our logistic relationship (described above). We then assumed that cumulative GHG emissions will increase linearly through time to determine when a 10% increase in RF is reached for each scenario.

Whereas Amstrup and Bitz^21^ calculated the uncertainty associated with their estimates, we only report the means because our goal was to show how the overall expected results vary depending on how different parameters are defined.

## Results

### Replication of Amstrup and Bitz methods

We initially compared our calculated number of IFD to those of Amstrup and Bitz^21^ based on their definitions of SIC, sea ice extent, and subpopulation boundaries to ensure we were accurately reproducing their results. Our results were largely consistent with exceptions in only 2 and 4 of the 42 years analyzed for the CS and SBS, respectively (Supplemental Methods S2). To better understand the cause of the discrepancies, we compared our calculated daily sea ice extents (based on decision rules and definitions that matched those used by Amstrup and Bitz^21^) to those provided by Amstrup and Bitz^21^ in their supplemental materials (Supplemental Methods S2).

We found that our calculated daily sea ice extents matched those of Amstrup and Bitz^21^ and that the discrepancy in IFD was likely due to Amstrup and Bitz^21^ not consistently applying their rule for summing IFD within a year (Supplemental Methods S2). Therefore, for the remainder of the study, we use our replication of the Amstrup and Bitz^21^ results (i.e., those based on the same decision rules for SIC, sea ice extent, boundary, and regression framework as Amstrup and Bitz^21^ but differing in the number of IFD in 2 years in the SBS subpopulation and 4 years in the CS subpopulation) to make comparisons to results from the other 8 scenarios we developed from alternative sets of decision rules. We chose to use our derivation of IFD over those of Amstrup and Bitz^21^ because we wanted to ensure differences in the results were due to the choice of parameters rather than inconsistencies in how IFD was summed across years. We henceforth refer to this replication of the Amstrup and Bitz^21^ results as the ‘Amstrup and Bitz scenario’.

### Ice-free days

**Fig. 3:**
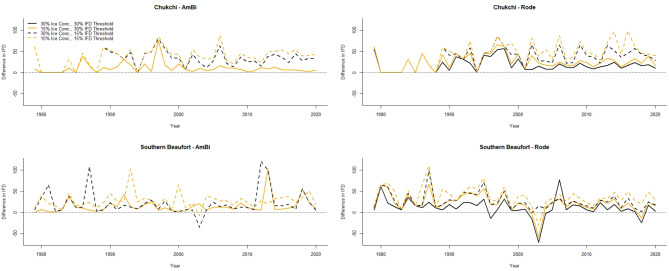
Difference in number of calculated ice-free days (IFD) between the Amstrup and Bitz scenario (i.e., using the boundary definition from Amstrup and Bitz^21^, a 30% sea ice concentration threshold to define sea ice extent, and a 30% IFD threshold) and the other scenarios we considered in our analysis. Values > 0 indicate higher estimates of IFD for the Amstrup and Bitz scenario than the scenario it is being compared to. There are no 30% ice extent or 30% IFD thresholds in the left panel plots because those represent the Amstrup and Bitz scenario that is being compared to the other scenarios. Chukchi refers to results for the Chukchi Sea subpopulation, and Southern Beaufort refers to results for the Southern Beaufort Sea subpopulation. Boundary definition is indicated by “AmBi” and “Rode” to indicate the boundary definitions used in Amstrup and Bitz^21^ and Rode et al.^23^, respectively.

We found large differences in the calculated number of IFD in the scenarios we considered compared to the Amstrup and Bitz scenario (Fig. 3; Tables 2, S1). Results for the CS subpopulation varied the most from the Amstrup and Bitz scenario, which had a higher number of IFD than the other scenarios in 35 years and an equivalent number of IFD in 7 years (Fig. 3). We note that the only years when the calculated IFD from our scenarios were equivalent to those of Amstrup and Bitz^21^ were when 0 IFD were calculated. In some years (i.e., 1997 and 2007), the number of IFD in the Amstrup and Bitz scenario was > 80 days higher than other scenarios (Fig. 3). Definitions of SIC, sea ice extent, and boundary that differed from those used by Amstrup and Bitz^21^ all led to reductions in the calculated number of IFD which results in positive values when subtracting the results from our scenarios from the Amstrup and Bitz scenario (Fig. 3). The results of Amstrup and Bitz^21^ for the CS subpopulation appeared most sensitive to how sea ice extent was defined, and which definition of subpopulation boundary is used (Table 2). In the CS, none of the scenarios led to the calculated number of annual IFD being greater than the Amstrup and Bitz scenario (Fig. 3).

**Table 2:** Mean number of ice-free days (IFD) between 1979 and 2020 for the Chukchi Sea (CS) and Southern Beaufort Sea (SBS) subpopulations (subpop) based on combinations of two different values of sea ice concentration (SIC) used to define sea ice extent (i.e., < 15% or < 30% SIC), two different definitions of the threshold used to define an IFD (i.e., < 15% or < 30% of 10-year March average sea ice concentration), and two delineations of the subpopulation boundaries (i.e., that used by Amstrup and Bitz^21^ and by Rode et al.^23^). The scenarios included in Amstrup and Bitz^21^ are depicted in bold.

Subpop	SIC (%)	IFD threshold (%)	Boundary	
			Amstrup and Bitz	Rode et al.
			$\:\stackrel{-}{x}$	$\:\stackrel{-}{x}$
CS	**30**	**30**	**68**	50
CS	30	15	38	32
CS	15	30	58	43
CS	15	15	32	25
SBS	**30**	**30**	**83**	71
SBS	30	15	61	55
SBS	15	30	68	60
SBS	15	15	55	45

We found similarly large differences in the number of IFD calculated for the SBS subpopulation for the different scenarios compared to the Amstrup and Bitz scenario (Fig. 3; Tables 2, S1). While the magnitude of difference in calculated IFD between the Amstrup and Bitz scenario and the other scenarios we considered was lower than for the CS subpopulation, there were still years when the calculated IFD for the Amstrup and Bitz scenario was > 100 compared to the other scenarios (Fig. 3). For example, in the scenario using the Amstrup and Bitz^21^ SBS boundary with sea ice extent defined by 30% SIC and a 15% IFD threshold, there were two years (i.e., 1987 and 2012) where the Amstrup and Bitz scenario led to > 100 day difference in the calculated number of IFD. We also observed some years when the calculated IFD were lower for the Amstrup and Bitz scenario compared to other scenarios we considered (Fig. 3). The scenario using the Rode et al.^23^-defined boundary with sea ice extent defined by 30% SIC and a 30% IFD threshold resulted in a lower calculated number of IFD in 5 years (i.e., 1996, 2002, 2003, 2004, and 2018) compared to the Amstrup and Bitz scenario. Similar to results for the CS subpopulation, calculated IFD appeared to be most sensitive to how sea ice extent was defined (Table 2). Conversely, choice in subpopulation boundary was less impactful for the SBS subpopulation than for the CS subpopulation (Table 3).

**Table 3:** Cumulative greenhouse gas emissions (GHG; see Supplemental methods S1 for how Amstrup and Bitz^21^ derived these data) estimated to result in a 10% increase recruitment failure (RF) and the year in which that threshold is projected to be crossed for polar bears in the Chukchi Sea (CS) and Southern Beaufort Sea (SBS) subpopulations based on combinations of two different values of sea ice concentration (SIC) used to define sea ice extent (i.e., < 15% or < 30% sea ice concentration), two different definitions of the threshold used to define an ice-free day (IFD; i.e., < 15% or < 30% of 10-year March average sea ice concentration), and two delineations of the subpopulation boundaries (i.e., that used by Amstrup and Bitz^21^ and by Rode et al.^23^) for both linear and logistic curves fit to the relationship between RF and GHG described in Molnár et al.^16^. The scenarios included in Amstrup and Bitz^21^ is depicted in bold. Subpopulation boundaries as defined by Amstrup and Bitz^21^ are identified as “AmBi” and boundaries defined by Rode et al.^23^ are identified as “Rode”.

Subpop	SIC	IFD	Boundary	Relationship between RF and GHG			
				Linear		Logistic	
				GHG	Year	GHG	Year
CS	**30**	**30**	**AmBi**	**1**,**521**	**2015**	**1**,**609**	**2017**
CS	30	15	AmBi	2,049	2029	2,145	2031
CS	15	30	AmBi	1,642	2017	1,727	2019
CS	15	15	AmBi	2,345	2036	2,456	2038
CS	30	30	Rode	1,752	2021	1,837	2023
CS	30	15	Rode	2,319	2035	2,429	2038
CS	15	30	Rode	1,906	2025	1,996	2027
CS	15	15	Rode	2,830	2047	2,966	2051
SBS	**30**	**30**	**AmBi**	**1**,**684**	**2018**	**1**,**834**	**2023**
SBS	30	15	AmBi	2,314	2035	2,479	2039
SBS	15	30	AmBi	2,367	2036	2,559	2041
SBS	15	15	AmBi	2,447	2038	2,609	2042
SBS	30	30	Rode	1,727	2019	1,848	2024
SBS	30	15	Rode	1,965	2027	2,079	2029
SBS	15	30	Rode	1,854	2024	1,967	2027
SBS	15	15	Rode	2,197	2032	2,316	2035

### Recruitment impact threshold

Following Amstrup and Bitz^21^, we calculated the first year that the CS and SBS subpopulations crossed the recruitment impact threshold of > 117 IFD in 3 of 5 consecutive years.

This was reported to occur in 2015 for the CS subpopulation and 2016 for the SBS subpopulation by Amstrup and Bitz^21^. We found, however, that the SBS subpopulation never reached the recruitment impact threshold between 1979 and 2020 for all scenarios we considered. For the CS subpopulation, only one scenario crossed the recruitment impact threshold, with that crossing estimated to have occurred in 2015. The decision rules for that scenario only differed from Amstrup and Bitz^21^ in using a 15% SIC threshold compared to their use of a 30% SIC threshold.

### Recruitment failure

The level of cumulative GHG that led to a 10% increase in RF varied considerably across scenarios for each subpopulation. For the CS subpopulation, the cumulative level of GHG emissions leading to a 10% increase in RF ranged from 1,521 to 2,966 Gt across scenarios (Table 3). The expected year a 10% increase in RF could occur ranged from 2015 to 2051 depending on the scenario (Table 3). Treating the relationship between RF and GHG as a logistic function vs. a linear function led to significantly higher levels of cumulative GHG (logistic: $\:\stackrel{-}{x}=\:2146\:Gt,\:SD=\:452$; linear: $\:\stackrel{-}{x}=2046,\:SD=435$ ) required to reach the 10% RF threshold (paired t-test: *t*_*7*_=-15.8; *p* < 0.001). Using the logistic function to model the relationship between RF and GHG emissions led to the 10% RF threshold being reached 2 to 4 years later than when modeling the relationship as linear (Table 3). Of all the scenarios considered for the CS subpopulation, the Amstrup and Bitz scenario had the lowest levels of cumulative GHG required to reach a 10% increase in RF and crossed the threshold earlier (Table 3).

The results for the SBS subpopulation were similar to those for the CS subpopulation. The cumulative level of GHG emissions leading to a 10% increase in RF ranged from 1,684 to 2,609 Gt across scenarios (Table 3). The expected year a 10% increase in RF could occur ranged from 2018 to 2042 depending on the scenario (Table 3). Treating the relationship between RF and GHG as a logistic function vs. a linear function led to significantly higher levels of cumulative GHG (logistic: $\:\stackrel{-}{x}=\:2212,\:SD=\:319$; linear: $\:\stackrel{-}{x}=2069,\:SD=300$) required to reach the 10% RF threshold (paired t-test: *t*_*7*_=-13.5; *p* < 0.001). Using the logistic function to model the relationship between RF and GHG emissions led to the 10% RF threshold being reached 2 to 7 years later than when modeling the relationship as linear (Table 3). Of all the scenarios considered for the SBS subpopulation, the Amstrup and Bitz scenario had the lowest levels of cumulative GHG required to reach a 10% increase in RF and crossed the threshold earlier (Table 3).

## Discussion

The approach developed by Amstrup and Bitz^21^ was an attempt to demonstrate that GHG emissions can be quantitatively linked to polar bear demography, thus allowing wildlife managers and policy makers to attribute specific levels of emissions to specific demographic effects. Previous research has shown that sea ice loss, caused by increasing GHG levels, has led to declines in polar bear reproductive success^18^. Thus, we agree that such a linkage exists, however, their approach relies entirely on relationships established for the WHB subpopulation which is likely not representative of most polar bear subpopulations. Indeed, subpopulations experience different sea ice dynamics^6^ which is reflected in other studies finding different sea ice thresholds at which population effects occur^9,10^.

Attempting to attribute cumulative GHG emissions to specific demographic responses of polar bears requires decision rules that are population-specific or model outputs that show minimal variation when different decisions rules are applied. We found large differences in calculated IFD, when the recruitment impact threshold is crossed, and when RF is expected to rise to 10% of reproductive females based solely on changes to decision rules and modeling decisions (i.e., linear vs. logistic relationships between FD and cumulative GHG emissions) for both subpopulations we studied. The decision rules and parameterizations we analyzed were based on other studies in the literature and specific to the subpopulations we studied^9,10^. Our results show how sensitive this relationship between GHG emissions and polar bear demography is to variation in decision rules and model parameterization. This suggests that the relationships involved are complex and presently not understood with the certainty necessary to facilitate the direct attribution of emissions to changes in polar bear recruitment.

For the CS and SBS subpopulations, our analysis indicates that results of Amstrup and Bitz^21^ are most sensitive to the choice of IFD threshold. Applying a 15% IFD threshold versus a 30% threshold (as in Amstrup and Bitz^21^) led to large declines in the anticipated number of FD (Fig. 3) and IFD (Table 2) for both subpopulations. For the CS subpopulation, the model was more sensitive to subpopulation boundary delineation than the choice of sea ice concentration threshold used to define sea ice extent (Table 2; Fig. 3). For the SBS, choice of subpopulation boundary was less impactful than for the CS subpopulation (Table 3), likely because Amstrup and Bitz^21^ had modified the SBS subpopulation boundary from Amstrup et al.^6^ to reflect only shallow (< 300 m depth) water over the continental shelf, leading to minimal differences in the extents of the subpopulation boundaries (Fig. 1). As a result, for the SBS subpopulation, model results were more sensitive to the choice of sea ice concentration used to define sea ice extent rather than subpopulation boundary delineation (Table 2; Fig. 3).

When considering the level of cumulative GHG emissions associated with 10% RF, the CS subpopulation was again most sensitive to IFD threshold, followed by choice of boundary delineation, sea ice extent, and the choice to model the relationship between RF and cumulative GHG emissions with a logistic curve rather than linear (Table 3). This differed from the SBS subpopulation, which showed the greatest sensitivity to sea ice extent threshold, followed by IFD threshold, treating the relationship between RF and cumulative GHG emissions as fitting a logistic curve, and lastly choice of subpopulation boundary delineation (Table 3).

Many studies have documented ecological relationships between polar bears and sea ice concentration^31^, however, factors other than sea ice concentration (or metrics derived from it) affect polar bear ecology. For example, Rode et al.^20^ showed that even though polar bears in the CS subpopulation experienced similar rates of summer sea ice loss as the SBS subpopulation, they did not appear to experience the same level of negative effects from sea ice loss as the SBS. This discrepancy was attributed to differences in the amount of time sea ice was over the productive continental shelf for both subpopulations^20^. Other studies have shown the importance of spatial variation in sea ice concentration in understanding polar bear space use patterns^31,32^ likely related to ice conditions conducive to hunting. Indeed, Rode et al.^33^ found positive relationships between polar bear body condition and recruitment with ringed (*Pusa hispida*) and bearded (*Erignathus barbatus*) seal body condition. Further, Rode et al.^33 ^found that sea ice conditions either had no effect on CS polar bear body condition and recruitment, or the relationship indicated increases in these metrics with declining sea ice. They concluded that sea ice is likely not currently a limiting factor for polar bears in the CS subpopulation^33^, which contrasts the estimate of Amstrup and Bitz^21^ that the recruitment impact threshold was crossed in the CS subpopulation in 2015.

Further complicating the use of sea ice concentration as a metric for estimating fasting duration of polar bears is the fact that sea ice concentration data can be inaccurate (with up to a 25% bias) during the summer melt period because the sensors cannot distinguish between open water and melt ponds on top of pack ice^34^. Kern et al.^34^ stated that it is extremely difficult to derive sea ice concentration of melting sea ice, which corresponds to the period when most ice-free days accumulate for polar bears. These limitations are likely not problematic when using SIC data to find relationships between space use or resource selection. However, when they are used to define when sea ice is assumed to be absent or at levels below which polar bears can effectively use for hunting, inferences may become biased. Thus, models attempting to link polar bear demographics to GHG emissions based on metrics solely related to sea ice concentration are likely overly simplistic. Even Amstrup and Bitz^8^ acknowledge that their approach requires data on subpopulation-specific polar bear body mass when they arrive on shore in autumn to be applicable across subpopulations. Relying on metrics derived from sea ice concentration alone is therefore not sufficient for attributing demographic responses to sea ice loss.

The challenges of assessing demographic responses to sea ice loss based on sea ice concentration metrics alone are highlighted by the results of Amstrup and Bitz^8^ for the CS subpopulation. In their analysis, Amstrup and Bitz^8^ found the CS subpopulation to be the most sensitive to GHG emissions of all polar bear subpopulations. This does not align with published studies that show the subpopulation has responded to sea ice loss better than the SBS subpopulation^14,20^ and that current demographic rates^29^ can support a robust subsistence harvest with low risk to subpopulation persistence^35^. We found that under all but one of the scenarios we considered the CS subpopulation did not reach the critical 117-day IFD threshold identified by Amstrup and Bitz^8^ and required higher levels of cumulative GHG emissions to reach a 10% increase in recruitment failure than the Amstrup and Bitz scenario. Given that the CS subpopulation is likely stable and healthy based on multiple lines of research^14,20,29,33,35,36^, our results suggest that the decision rules Amstrup and Bitz^8^ used do not reflect the drivers of demographic change for the CS subpopulation. These results demonstrate the need for subpopulation-specific parameterizations when attempting to link sea ice metrics to reproductive success and GHG emissions.

We were surprised that no scenario for the SBS subpopulation suggested that RF had occurred at high levels or that the subpopulation had crossed the 117-day recruitment impact threshold given observed declines in subpopulation abundance over the past 25 years^17,37,38^, increased rates of spring fasting^14^, and increased periods of land use and the proportion of the population using land in summer and autumn^9^. This might reflect that the observed declines in polar bear demography are more attributable to declines in prey abundance than reductions in sea ice, as suggested by Rode et al.^11^. Thus, population declines might not be primarily driven by increased land use which is the primary mechanism Amstrup and Bitz^21^ assume drives documented declines in polar bear demography. Further, it might also be possible that polar bears in the SBS have increased use of terrestrial food resources while onshore in autumn. It was noted by Bromaghin et al.^38^ that bowhead whale remains from subsistence harvest^39^ could partially offset lost hunting opportunities associated with longer durations on shore. These remains have been shown to contribute substantial energetic resources to polar bears^40^ and their positive contributions to bears in the SBS subpopulation would remain obscured by relying solely on sea ice concentration metrics to identify periods of fasting as presented by Amstrup and Bitz^21^. Thus, myriad factors likely affect polar bear demographics beyond sea ice loss alone.

Our study is not meant as an exhaustive assessment of the sensitivity of the Amstrup and Bitz^21^ model to all decision rules and parameters they used. Rather, we focused on key parameters we believed the approach would be most sensitive to and where alternative definitions exist with support in the literature. If the approach by Amstrup and Bitz^21^ is to be applied, additional work is needed to determine how sensitive it is to additional assumptions (e.g., no energy derived from terrestrial foods while “fasting” on shore) and decision rules (e.g., how the number of IFD is calculated annually; Supplemental Methods S2).

Our results indicate that further refinement of the Amstrup and Bitz^21^ approach is needed before it can be relied upon to precisely attribute specific levels of GHG emissions to reductions in polar bear demographic rates. Our results suggest that the approach is too sensitive to the choice of decision and modeling assumptions to evaluate the impacts of GHG emissions on polar bear demographic rates. We agree with the suggestions of Amstrup and Bitz^21^ that an approach that relies more directly on polar bear energetics, and is parameterized specifically for the subpopulation of interest, is likely necessary before cumulative GHG emissions can be accurately attributed to declines in polar bear demographics. Thus, to meet the ultimate objectives of Amstrup and Bitz^21^, researchers must establish subpopulation-specific rates of energetic loss during fasting (e.g., Pagano et al.^41^), subpopulation-specific estimates of polar bear body mass entering a period of fasting, and more objective metrics than sea ice concentration for establishing when polar bears enter a period of fasting (e.g., activity data obtained from collared bears; e.g. Ware et al.^15^).

## Electronic supplementary material

Below is the link to the electronic supplementary material.

Supplementary Material 1

Supplementary Material 2

Supplementary Material 3

Supplementary Material 4

## Data Availability

Data is provided within the manuscript or supplementary information files.
