# Supplementary material for: Model sensitivity limits attribution of greenhouse gas emissions to polar bear demographic rates
Source: Sci Rep. 2025 Feb 10;15:4975. doi: 10.1038/s41598-025-89218-3 (PMC11811060; doi:10.1038/s41598-025-89218-3)
Supplement: Supplementary file 1 — Supplementary Material 1 [file 41598_2025_89218_MOESM1_ESM.docx]

**Supplemental Materials S1:** Replicating Amstrup and Bitz methods

A large portion of the methods Amstrup and Bitz^1^ used for their analysis was only found in the MATLAB code provided in their supplemental materials. Therefore, we provide a detailed description of the methods and R code (Supplemental Materials S3) we employed so they are easier to replicate.

We obtained daily passive microwave sea ice concentration data from the period 1979 – 2020^2^. We used version 2 of these data which were released in 2022. While Amstrup and Bitz^1^ used version 1 data for their analysis, they also repeated their analysis based on the version 2 data and found the results to be identical. We determined which sea ice concentration (SIC) grid cells were located within the ranges of the Chukchi Sea (CS) or the Southern Beaufort Sea (SBS) subpopulation as defined in Amstrup et al.^3^. Following Amstrup and Bitz^1^, we restricted grid cells in the SBS to those that were in waters over the continental shelf (i.e., < 300 m depth). Even though SIC grid cells are represented as 252 km^2^ cells, each cell actually represents a different sized area (https://nsidc.org/data/user-resources/help-center/guide-nsidcs-polar-stereographic-projection; accessed 21 Aug 2024). We therefore obtained the grid cell-specific areas (https://github.com/NOAA-Strausz/EcoFOCI_ssmi_ice/blob/master/psn25area_v3.dat; accessed 21 Aug 2024) which we used to determine daily sea ice extent. For each day between 1979 – 2020 we determined which grid cells had SIC > 30% and summed the areas represented by these cells to define daily sea ice extents for the CS and SBS subpopulations. While Amstrup and Bitz^1^ excluded leap-days (i.e., 29 Feb) when they occurred in their time series, we retained them because we did not want to exclude data.

The majority of the SIC dataset was available daily, however, between 1979 and 1987, data were only available every-other day. Following Amstrup and Bitz^1^, we linearly interpolated the missing values of daily sea ice extent as follows: $e_{i}=\left( e_{i-1}+e_{i+1} \right)/2$, where *e_i_* is the sea ice extent on day *i*, and *e_i-i_* and *e_i+1_* are sea ice extents the day before and after day *i*, respectively.

To calculate the number of “ice-free” days (IFD) polar bears experienced, Amstrup and Bitz^1^ determined a reference sea ice extent threshold, below which it is assumed the energy acquired through hunting is outweighed by the costs of acquiring prey^4^. The reference sea ice extent was calculated as 30% of the average daily March sea ice extents (as defined above) between 1979 – 1988 for the CS and SBS subpopulations. We then determined which days between 1979 – 2020 had sea ice extents less than the IFD threshold for both subpopulations.

To determine the total number of IFD for a given subpopulation in a given year, Amstrup and Bitz^1^ defined the start and end of the “ice-free” period based on a decision rule (found in their supplemental model code). Specifically, the decision rule defined the start of the “ice-free” period as the first day when there are 3 consecutive days where sea ice extent is less than the reference extent (as defined above). Similarly, they defined the end of the “ice-free” period as the first date when there were 3 consecutive days when sea ice extent was above the reference extent. The number of IFD was then defined as the number of days between the calculated start and end dates.

In their analysis, Amstrup and Bitz^1^ treated the number of IFD as being equivalent to the number of fast days (FD) polar bears experienced in the CS and SBS subpopulations and linearly modeled the relationship between FD and the cumulative GHG through time; $FD= \gamma_{0}+ \gamma_{1}GHG$ where γ are the modeled regression coefficients. We used the annual cumulative GHG values provided by Amstrup and Bitz^1^ to model this relationship for both subpopulations in our analysis using the ‘lm’ function in R^5^. Amstrup and Bitz^1^ created their GHG dataset by integrating two datasets. The first obtained “Kyoto” greenhouse gassed (i.e., CO2, CH4, N2O and fluorinated gases) while excluding emissions from land use and forest changes from Gütschow et al.^6^ They then added emissions from land use and forest changes based on data from Friedlingstein et al.^7^

The framework proposed by Amstrup and Bitz^1^ used data from Molnár et al.^8^ to calculate a linear relationship between FD and the percent of lactating females with cubs that exhibit recruitment failure (RF). We obtained the same dataset used by Amstrup and Bitz^1^ from Peter Molnár (pers. comm) and calculated the linear relationship between FD and recruitment failure with the ‘lm’ function in R^5^; $RF= \beta_{0}+ \beta_{1}FD$, where β are the modeled regression coefficients.

Finally, as in Amstrup and Bitz^1^, we combined the regression results from the two linear models described above to establish a quantitative relationship between GHG and RF. This was accomplished by substituting the observed FD in the RF model with the regression equation obtained from the modeled relationship between FD and GHG. This substitution took the following form: $RF=$ $\beta_{0}+ \beta_{1}( \gamma_{0}+ \gamma_{1}GHG)$.

**Literature Cited**

1. Amstrup, S. C. & Bitz, C. M. Unlock the Endangered Species Act to address GHG emissions. *Science (1979)* **381**, 949–951 (2023).

2. DiGirolamo, N. E., Parkinson, C. L., Cavalieri, D. J., Gloersen, P. & Zwally, H. J. *Updated Yearly Sea Ice Concentrations from Nimbus-7 SMMR and DMSP SSM/I-SSMIS Passive Microwave Data, Version 2*. (2022).

3. Amstrup, S. C., Marcot, B. G. & Douglas, D. C. A Bayesian network modeling approach to forecasting the 21st century worldwide status of polar bears. *Geophysical Monograph Series* **180**, 213–268 (2008).

4. Cherry, S. G., Derocher, A. E., Thiemann, G. W. & Lunn, N. J. Migration phenology and seasonal fidelity of an Arctic marine predator in relation to sea ice dynamics. *Journal of Animal Ecology* **82**, 912–921 (2013).

5. R Development Core Team. *R: A Language and Environment for Statistical Computing*. (Foundation for Statistical Computing, Vienna, Austria, 2023).

6. Gütschow, J., Günther, A. & Pflüger, M. *The PRIMAP-Hist National Historical Emissions Time Series (1750 - 2019). v.2.3.1.* (2021).

7. Friedlingstein, P. *et al.* Global Carbon Budget 2021. *Earth Syst Sci Data* **14**, 1917–2005 (2022).

8. Molnár, P. K. *et al.* Fasting season length sets temporal limits for global polar bear persistence. *Nat Clim Chang* **10**, 732–738 (2020).
