# Supplementary material for: Model sensitivity limits attribution of greenhouse gas emissions to polar bear demographic rates
Source: Sci Rep. 2025 Feb 10;15:4975. doi: 10.1038/s41598-025-89218-3 (PMC11811060; doi:10.1038/s41598-025-89218-3)
Supplement: Supplementary file 2 — Supplementary Material 2 [file 41598_2025_89218_MOESM2_ESM.docx]

**Supplemental Materials S3**. Model code and objects needed to reproduce results from the paper.

The supplemental files contain the R script to run the analysis, “*analysis_code.R*” and the R workspace “*analysis_workspace.RData*” with study outputs and objects required to reproduce our results.

The R workspace contains the following objects:

*concentration.results*: Derived daily sea ice extents for each scenario considered.

*cs.pbsg*: Chukchi Sea subpopulation boundary used by Amstrup and Bitz (2023).

*cs.rode*: Chukchi Sea subpopulation boundary used by Rode et al. (2022).

*sbs.pbsg*: Southern Beaufort Sea subpopulation boundary used by Amstrup and Bitz (2023).

*sbs.rode*: Southern Beaufort Sea subpopulation boundary used by Rode et al. (2022).

*raster.xy*: Pixel centroid coordinates (x, y; NSIDC NPStereographic projection) for sea ice raster. Includes whether pixel occurs in each subpopulation boundary, and the area represented by the pixel.

*rec.failure*: Output from neg.recruit.func. Provides calculated cumulative GHG emissions required to reach 10% RF and the year this likely occurs.

*ref.extent*: Output of calculated reference extents for calculating ice-free-days.

*summary.IFD*: Output of calculated ice-free-days under the different scenarios between 1979 and 2020.

*neg.recruit*: A function for calculating when 10% RF is reached for each scenario and the predicted year when that is expected to occur.

*start.end*: A function for calculating the start and end of the ice-free season annually.

*pix.area*: Raster with pixel values based on area represented by each pixel
